# The ethics of N of 1 trials in routine practice and other problems of publication ethics

**DOI:** 10.1186/1757-1626-1-78

**Published:** 2008-08-08

**Authors:** Richard Smith

**Affiliations:** 1 Editor-in-Chief, Cases Journal, Middlesex House, 34-42 Cleveland Street, London, W1T 4LB, UK

The bias of *Cases Journal *is to publish unless there are strong reasons why we can't. Bruce Arroll and colleagues tell an interesting story[1] of how neighbours helped two couples conduct single patient trials to determine whether their babies had milk allergy. The story is informative and well told but raises several problems, most of them ethical, that we've had to think through before publishing.

Firstly, the authors have no consent for publication from the patients. I spent years struggling with the problem of patient consent when I was editor of the BMJ, and we moved from a position like all other journals of never seeking consent to insisting on consent in all cases. The latter proved to be too extreme a position, particularly for anecdotes from years ago – which we published in the BMJ and were popular. BMJ policy now requires four conditions to be met for a story to be told about a patient without consent. The conditions are:

a) The patient is dead and his or her family is untraceable to seek consent from

(b) The article contains a worthwhile clinical lesson or public health point which could not be as effectively made in any other way.

(c) A reasonable person in the position of the patient's relatives would not be expected to object to the publication of the case.

(d) The risk of identification of the patient is minimised by measures designed to prevent the identity of the patient being revealed either to others or to the patient's relatives.

It's most unlikely that either the parents or the babies are dead – so the first condition is not met. The article makes an excellent point, but it could probably be made in another way – and possibly has been. So we can't be confident that the second condition is met. The third and fourth conditions are, however, met handsomely, and I find it very hard to imagine the parents or the babies being upset by publication of this report – even in the highly unlikely event that they could be recognised. I'm thus happy to publish despite all four conditions not being met.

A second problem is that the babies were given soya-based milk on unconvincing grounds and could have suffered an anaphylactic reaction. The reviewer raised this point, and the authors acknowledge the risk – but dismiss it as small. The patients didn't suffer an anaphylactic reaction, and it would seem absurdly heavy handed to decline to publish an interesting story in unknown patients on the grounds that the patients ran a small risk 20 years ago.

A third problem is that we have lots of missing data. We can't see the charts of when the babies were given the different milks and how the timings related to the pattern of symptoms. Nor do we have any follow up data on the babies. We don't even know if the baby who did appear to have cow's milk allergy did better when cow's milk was removed from the child's diet. Nor do we even know the genders of the babies. It would be good to know all of these things, but I judge that the story still has interest and importance without those details.

Fourthly, the doctor conducted an experiment in two babies without consent from an ethics committee (or institutional review board). Can this be acceptable? Did the parents understand that there babies were included in a experiment? They must have done: the whole point of the exercise was that it was an experiment. The reality of 20 years ago in most, perhaps all, countries is that there were no ethics committees for primary care research. Indeed, there wasn't much research in primary care. So again it would seem slightly absurd to decline to publish the paper because of the failure to get ethics committee approval.

But what if a doctor were to want to conduct such a trial today? Should he or she go ahead without ethics committee approval? Gordon Guyatt, one of the authors of the study, has tackled this questions with others. [2] They argue that if the study is undertaken as part of clinical practice then there is no need for consent from an ethics committee, but the doctors should ensure that the patients understand fully what is happening and get written consent. If, however, the study us undertaken as part of research then approval from an ethics committee is essential.

I'm uncertain exactly how you tell whether something is conducted for clinical or research reasons – perhaps the distinction is that one is conducted for the benefit of the patients and the other more for the benefit of the broader community. This doesn't seem to me to be an entirely clear distinction, especially if, as in this case, an experiment conducted for clinical reasons is published.

I can only think that it would be wise to contact the chair of the local committee. Ethics committees famously vary in their judgements. I tend to agree with Guyatt and colleagues that an N of 1 trial that is happening as part of routine practice would not need consent from an ethics committee. The patients should, of course, give fully informed consent – preferably in writing – but the nature of much of medical practice is that doctors and patients are constantly "experimenting" to discover, for example, "whether time will sort this out" or "whether this drug might work better than the one you are on at the moment." It would be clearly impractical to consult an ethics committee over every such experiment. The differences with the "experiments" described here are that they are being published and ironically are well designed – with randomisation and controls. They thus feel much more like experiments than the experiments that happen every day in many meetings between doctors and patients.

So this paper not only tells an interesting story attractively but has also thrown up enough questions to prompt an editorial longer than the paper – for which perhaps I ought to apologise.
